# Trastuzumab distribution in an *in-vivo* and *in-vitro* model of brain metastases of breast cancer

**DOI:** 10.18632/oncotarget.19634

**Published:** 2017-07-26

**Authors:** Tori B. Terrell-Hall, Mohamed Ismail Nounou, Fatema El-Amrawy, Jessica I.G. Griffith, Paul R. Lockman

**Affiliations:** ^1^ Department of Basic Pharmaceutical Sciences, School of Pharmacy, West Virginia University HSC, Morgantown, West Virginia 26506, USA; ^2^ Department of Pharmaceutics, Faculty of Pharmacy, Alexandria University, Alexandria 21521, Egypt; ^3^ Department of Pharmaceutical Sciences, School of Pharmacy, University of Saint Joseph (USJ), Hartford, Connecticut 06103, USA

**Keywords:** drug delivery, metastasis, microfluidic device, blood brain barrier, permeability

## Abstract

**Background:**

Drug and antibody delivery to brain metastases has been highly debated in the literature. The blood-tumor barrier (BTB) is more permeable than the blood-brain barrier (BBB), and has shown to have highly functioning efflux transporters and barrier properties, which limits delivery of targeted therapies.

**Methods:**

We characterized the permeability of ^125^I-trastuzumab in an *in-vivo*, and fluorescent trastuzumab-Rhodamine123 (t-Rho123) in a novel microfluidic *in-vitro*, BBB and BTB brain metastases of breast cancer model. *In-vivo*: Human MDA-MB-231-HER2+ metastatic breast cancer cells were grown and maintained under static conditions. Cells were harvested at 80% confluency and prepped for intra-cardiac injection into 20 homozygous female Nu/Nu mice. *In-vitro*: In a microfluidic device (SynVivo), human umbilical vein endothelial cells were grown and maintained under shear stress conditions in the outer compartment and co-cultured with CTX-TNA2 rat brain astrocytes (BBB) or Met-1 metastatic HER2+ murine breast cancer cells (BTB), which were maintained in the central compartment under static conditions.

**Results:**

Tissue distribution of ^125^I-trastuzumab revealed only ~3% of injected dose reached normal brain, with ~5% of injected dose reaching brain tumors. No clear correlation was observed between size of metastases and the amount of ^125^I-trastuzumab localized *in-vivo*. This heterogeneity was paralleled *in-vitro*, where the distribution of t-Rho123 from the outer chamber to the central chamber of the microfluidic device was qualitatively and quantitatively analyzed over time. The rate of t-Rho123 linear uptake in the BBB (0.27 ± 0.33 × 10^4^) and BTB (1.29 ± 0.93 × 10^4^) showed to be significantly greater than 0 (p < 0.05). The BTB devices showed significant heterogenetic tendencies, as seen in *in-vivo*.

**Conclusions:**

This study is one of the first studies to measure antibody movement across the blood-brain and blood-tumor barriers, and demonstrates that, though in small and most likely not efficacious quantities, trastuzumab does cross the blood-brain and blood-tumor barriers.

## INTRODUCTION

Brain metastases are a fatal neurological complication of breast cancer which have historically been a major cause of morbidity. Women with symptomatic central nervous system (CNS) metastases have a median survival of approximately 4 months [1]. Furthermore, less than 2% of women survive two years post-diagnosis [2]. The risk of developing brain metastasis has been reported to range from 10 to 16 % among advanced-stage breast cancer patients, making it the second most common cause of metastatic brain tumors after lung cancer (10–25%) [3–7].

Among the many associated risk factors in the development of brain metastases from breast cancer, hormone receptor status is significant [8]. Within the HER2-positive (HER2+) subset, hormone receptor status is associated with CNS relapse. Patients with hormone receptor-negative/HER2+ tumors experience increased risk of the CNS as site of first relapse as compared to patients with hormone receptor-positive/HER2+ tumors [9–12]. Up to 37% of patients with HER2+ breast cancer relapse is associated with intracranial metastases, despite control of the peripheral tumors [13–15]. Palmieri *et al.* demonstrated that HER2 overexpression increases the outgrowth of metastatic tumors cells in the brain in breast carcinoma cell lines [12]. A limiting factor in the treatment of brain metastases is the inability of chemotherapy to reach the desired tumor location. This is due, in large part, to the presence of a strictly controlled and complex vascular network known as the blood-brain barrier (BBB).

The BBB is a physical and functional barrier limiting passive diffusion of extrinsic agents into brain [16–19]. The BBB is mainly composed of endothelial cells, in addition to pericytes, astrocytes and neuronal cells that play an important supportive role in the function of the BBB [20]. The BBB endothelial cells are linked together by tight junction protein complexes, which prevent passive paracellular transport of most water-soluble compounds and many lipid soluble compounds, with the exception of small gaseous compounds like carbon dioxide and molecular water [16, 19–23].

The function and organization of the BBB may be altered under pathological conditions. In the case of tumors, the BBB's structure and integrity are altered, forming the “blood-tumor barrier” (BTB) [22]. The BTB differs from the BBB in its decreased tight junction expression [24], a disruption of the basement membrane [25] and an increase in permeability [26, 27]. However, radiologic data have shown that not all brain metastases display significantly elevated BTB permeability [28]. The changes in BTB vascular permeability are typically heterogeneous throughout the tumor region [29, 30]. It has been observed that brain metastases from HER2+ breast cancers infiltrate brain parenchyma without disrupting the BBB, unlike brain metastases from triple negative or basal-type breast cancers, which often disrupt the BBB [9, 14, 31].

Targeted therapies have modernized cancer treatment, offering an improved therapeutic ratio [32]. These drugs, such as small molecule inhibitors (lapatinib) [33] and monoclonal antibodies and their drug conjugates (mABs) [32], have prolonged progression-free survival and effected some reduction of CNS tumor burden in patients [34–36]. With the advent of these therapies, patients with HER2+ metastatic disease are now living 2-3 years post-diagnosis [36]. However, the ability of these drugs and antibodies to permeate and distribute within the brain and brain metastases has not completely been elucidated. Table 1 details some examples from literature of various antibody and antibody-drug conjugates’ permeability to the brain in preclinical models, with or without BBB disruption.

**Table 1:** Examples from literature of various antibody and antibody-drug conjugates’ permeability to the brain in preclinical models, with or without BBB disruption

Antibody	Ab use	Drug	Model	Efficacy	Ref.
^125^I-MAb	Tracer	-	Normal rat brain	Permeability was increased with osmotic BBB disruption.	Neuwelt et al., 1986 [52]
^125^I-IgG 96.5^125^I-FAb 96.5^125^I-FAb 48.7				· Permeability increased with osmotic BBB disruption.· Enhanced & sustained retention of IgG in the brain.	Neuwelt et al., 1987 [53]
L6 IgGF(Ab’)2FAb			LX-1 human small-cell lung carcinoma intracerebral xenograft in nude rat	Antibody permeability to the brain increased with increased delay in dosing	Neuwelt et al.,1994 [54]
L6 IgG-conjugated iron oxine nanoparticles	Imaging (MRI)			Specific antibody displayed specific binding and potential for diagnostic enhancement of MRI	Remsen et al., 1996 [55]
SGN-15 MAb	Therapy	Doxorubicin		Immunoconjugate delivered across the BBB was effective against antigen-positive tumor cells	Neuwelt et al., 2003 [56]
Bevacizumab		Carboplatin	UW28 human glioma xenografts in nude rats	Combination therapy of Bevacizumab with carboplatin was more effective than Bevacizumab alone.	Jahnke et al., 2009 [57]
RituximabAnti-CD20	Therapy & Imaging (MRI)	Rituximabanti-CD20	Lymphoma rat model	Rituximab was effective at decreasing tumor volume and improving survival rate in a CNS lymphoma model	Muldoon et al., 2011 [58]
TDM1	Therapy	Emantsine	Her2+ BT474 or MDA-MB-361 CNS metastases in nude mice	TDM1 displayed increased survival rate in comparison to trastuzumab	Askoxylakis et al., 2015 [46]
^89^Zr-trastuzumabmuMAb 4D5TDM1	Tracer& Therapy	Emantsine	HER2-expressing transgenic Fo2-1282 or Fo5 mouse breast cancer in mice	· Trastuzumab displayed preferential uptake in tumor lessions· muMAb 4D5 and TDM1 significantly increased survival, as did combination therapy with PI3K/mTOR inhibitor GNE-317	Phillips et al., 2017 [59]

In this work, we have tested the permeability of ^125^I-trastuzumab in an *in-vivo,* and fluorescent trastuzumab-Rho123 (t-Rho123) in a novel *in-vitro* model of brain metastases of breast cancer. This study demonstrates that trastuzumab crosses the BTB and the accumulates in tumor in our preclinical model of brain metastases of breast cancer and is accompanied by confirmatory microfluidic *in-vitro* experiments.

## RESULTS

**Figure 1:**
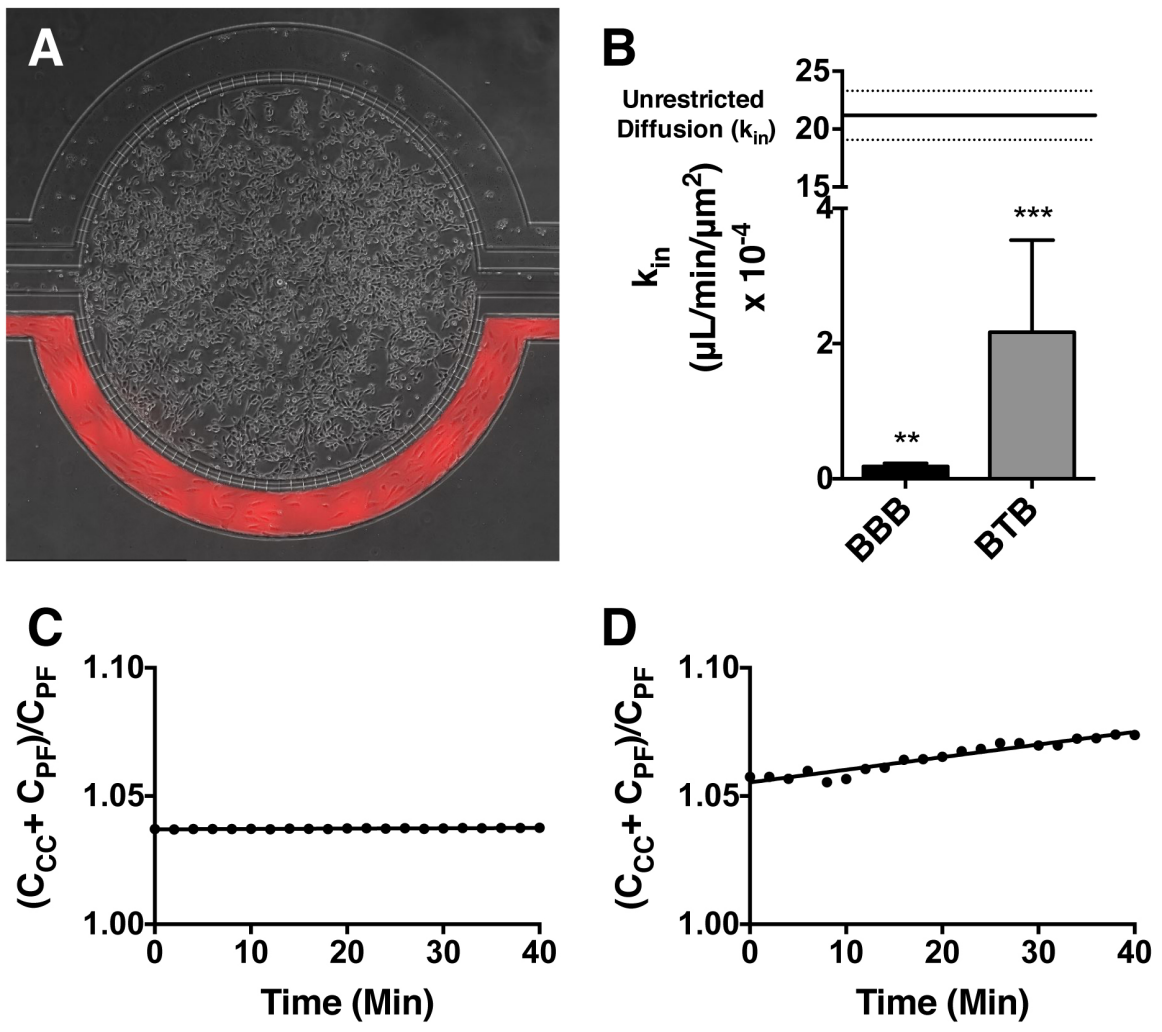
Mechanism of trastuzumab movement Linear central compartment accumulation of t-Rho123 in *in-vitro* BBB and BTB microfluidic chip models. Representative image of model with TRITC labeled t-Rho123 flowing over HUVEC cells in the outer compartment and either astrocytes or JIMT-1 cancer cells in the central compartment **(A)**. Rate of t-Rho123 movement in each model plotted against the unrestricted diffusion k_in_; ^**^ p<0.0033 significance between BBB model and unrestricted diffusion k_in_, n=3; ^***^ p<0.0005 significance between BTB model and unrestricted diffusion k_in_, n=3. All data represent mean ± S.E.M. Each model is significantly different than 0 (p < 0.05) **(B)**. Representative graphs of the rate of accumulation of t-Rho123 in the BBB **(C)** and BTB **(D)** microfluidic devices (n≥3).

To visualize *in-vitro* movement of t-Rho123, microfluidic BBB and BTB chips (Figure 1A) were established and utilized as previously published [37]. The distribution of t-Rho123 in BBB and BTB models was analyzed. We observed a linear increase of fluorescent trastuzumab uptake in both the BBB (0.27 ± 0.33 × 10^4^) (Figure 1C) and BTB (1.29 ± 0.93 × 10^4^) (Figure 1D) models significantly greater than 0 (p < 0.05). The rate of movement of fluorescent trastuzumab was quantified through the addition of a region of interest in the outer chamber (comparative to concentration of drug in plasma, C_PF_) and a region of interest in the central chamber (comparative to concentration of drug in brain, C_CC_), then divided by the sum intensity of tracer in the outer chamber (C_CC_ + C_PF_/ C_PF_) and plotted over time. The slope of this line, k_in_ (μL/min/μm^2^), was plotted and graphed (Figure 1B) as the mean ± S.E.M. for the BBB (0.18 ± 0.05, n=3) and BTB (2.12 ± 1.36, n=3) models. Both the k_in_ for the BBB models as well as the heterogeneity of the k_in_ values in the BTB models were comparable to *in-vivo*. The BBB (p<0.0033) and BTB (p<0.0005) models were significantly different in comparison to the unrestricted diffusion k_in_ of this model, as previously described [38].

**Figure 2:**
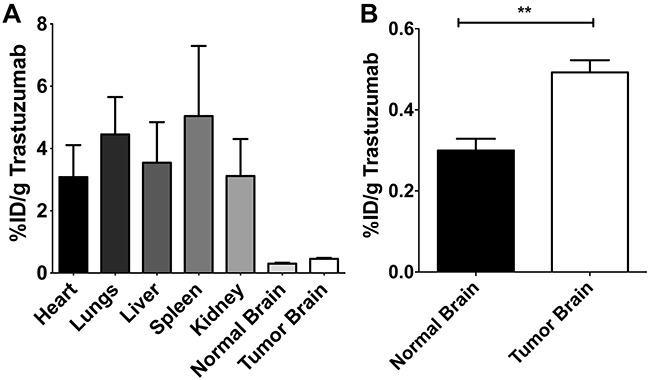
The distribution of radiolabeled ^125^I–trastuzumab in various body organs **(A)** and in normal and tumor brain tissues **(B)**. “%ID/g” refers to the percentage of injected dose of ^125^I-trastuzumab per gram of tissue.

Organ distribution of ^125^I-trastuzumab after intracardiac injection of 20 Nu/Nu mice with a HER2+ breast cancer cell line was determined. After the mice developed metastases (~32 days post-injection), iodinated-labeled ^125^I-trastuzumab was injected and allowed to circulate, followed by the administration of TRD 10 minutes prior to decapitation. Quantitative autoradiography (QAR) was used to measure the brain tissue distribution of ^125^I-trastuzumab. Figure 2A represents organ distribution of ^125^I-trastuzumab, variability in different body organs is observed. ^125^I-trastuzumab was found in significant quantities in spleen (5.04%, SD= 3.91), lungs (4.45%, SD= 2.08), liver (3.54%, SD= 2.26), kidney (3.12 %, SD= 2.06), and heart (3.08%, SD= 1.78) compared to normal brain (0.30%, SD= 0.22) and tumor brain tissues (0.46%, SD= 0.46). The accumulation of ^125^I-trastuzumab in tumor brain was 1.5 fold higher than normal brain tissue (p<0.0001) (Figure 2B).

**Figure 3:**
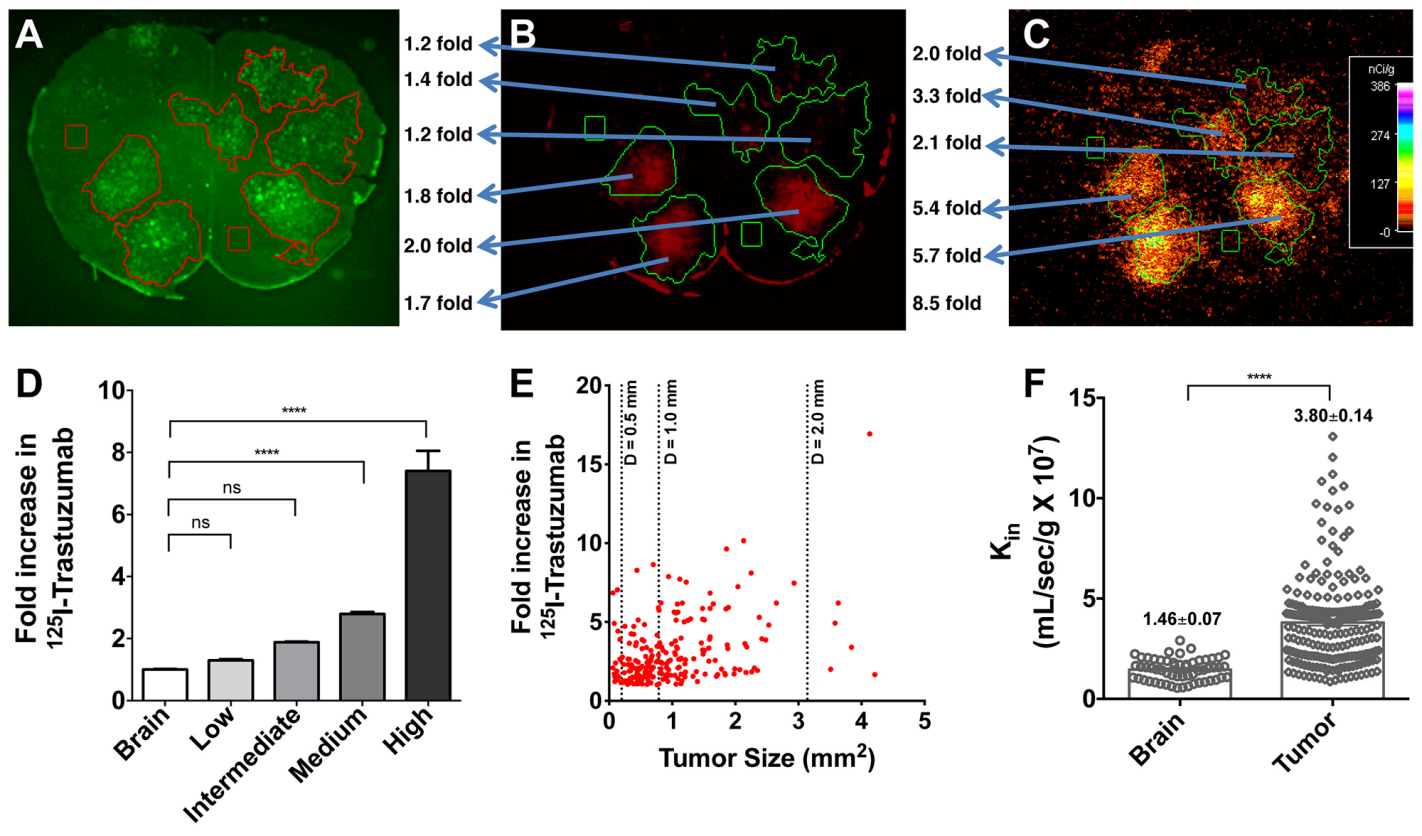
Heterogeneous and limited distribution of ^125^I-trastuzumab in preclinical brain metastases of breast cancer model Representative images of location of eGFP labeled 231-Br-Her2 brain metastases **(A)** and brain accumulation of Texas Red 625 Da **(B)** and ^125^I-trastuzumab **(C)** are shown. Metastases were categorized into four groups based upon the magnitude of permeability change compared to normal brain, where low, intermediate and high corresponds to the following: < mean brain + 3xSD; > mean brain + 3xSD but <2 fold; 2-4 fold; >4 fold, respectively (values represent mean ± SD, n=251 metastases) **(D)**. Fold increase in ^125^I-trastuzumab (over normal brain) was plotted versus metastasis size (mm^2^) in individual 231-Br-Her2 brain metastases. Metastases size was calculated based on tumor(s) surface area generated from the fluorescent tumor lesions in brain slices (3A) and reported in mm^2^ **(E)**. The correlation was minimal with ^125^I-trastuzumab fold increase versus lesion size. k_in_ values for normal and tumor areas of the brain **(F)**. At least, 20 slides/animal/group were analyzed in each study.

Heterogeneous and limited distribution of ^125^I-trastuzumab in our preclinical brain metastases of breast cancer model is shown in Figure 3A-3C. Metastases were categorized into four groups based upon the magnitude of permeability change compared to normal brain, where low, intermediate, medium and high corresponds to the following: < mean brain + 3xSD; > mean brain + 3xSD but < 2 fold; 2-4 fold; > 4 fold, respectively. The mean and standard deviation of the four groups were, 1.30 and 0.34 for low permeability, 1.88 and 0.07 for intermediate permeability, 2.79 and 0.61 for medium permeability, and 7.40 and 4.66 for high permeability (Figure 3D).

Fold increase in ^125^I-trastuzumab (over normal brain) was plotted versus metastasis size (mm^2^) in individual 231-Br-HER2 brain metastases (Figure 3E). No clear correlation was found between the size of brain metastases and the amount of ^125^I-trastuzumab localized within the tumor region (Figure 3E). K_in_ values were determined separately for normal and tumor areas of the brain (Figure 3F). Mean k_in_ for normal brain tissue was 1.457×10^7^ mL/sec/g (SD= 0.55) while mean k_in_ in the case of tumor brain was 3.80 mL/sec/g (SD= 2.17).

## DISCUSSION

Treatment of brain metastases of breast cancer conventionally consists of surgery, whole brain radiation, stereotactic radiosurgery, chemotherapy, and/or biological therapies [39]. Various chemotherapeutic agents have shown only a modest effect on survival due to their limited ability to cross the BBB [39]. In a preclinical study using two different models of brain metastases of breast cancer, most metastases exhibited some increased BTB permeability in comparison to normal brain. However, BTB permeability remained poorly correlated with lesion size, and only approximately 10% of lesions with the highest permeability exhibited cytotoxic responses to paclitaxel or doxorubicin [29]. In low-grade gliomas, the BTB resembles a normal functioning BBB, while in high-grade gliomas, BTB is disrupted “leaky”, as it is characterized by major alterations of the normal vascular function, shown through contrast-enhanced MRI [22, 40]. However, the magnitude of this local disruption may or may not be sufficient to allow drug penetration in meaningful quantities, and is thus considered a major obstacle for drug delivery to the brain [41].

Trastuzumab (Herceptin^®^, Genentech/ Roche), is a widely used humanized mAB for the treatment of HER2+ breast cancer due to its ability to recognize and bind to the extracellular juxtamembrane domain of HER2. Through this binding, trastuzumab is able to inhibit the proliferation, and therefore survival, of HER2-dependent tumors [7]. The ability of trastuzumab to significantly cross the BBB is unclear [42]. Şendur *et al.* [43] reported a case study using a combination of lapatinib and capecitabine followed by trastuzumab in HER2+ brain metastatic breast cancer. No progression of cranial metastases was found post-treatment. In another case series by Mutlu *et al.* [44], one in three patients with HER2+ breast cancer brain metastasis maintained the brain metastases post-treatment with a combination of weekly trastuzumab plus vinorelbine, however, these studies do not necessarily indicate the effectiveness of trastuzumab alone. In an *in-vivo* study by Kodack *et al.*, it was observed that through the use of a combination of a HER2 inhibitor with an anti–VEGF receptor-2 antibody, trastuzumab, and lapatinib, tumor growth was significantly slowed in the brain, resulting in increased survival in a mouse model of HER2-amplified breast cancer brain metastasis using an orthotopic xenograft of BT474 cells [45].

An antibody-drug conjugate of trastuzumab, trastuzumab-emantsine (TDM1), has displayed very promising efficacy in preclinical and clinical trials in CNS metastases [34, 46]. However, in patients without brain metastases, the ratio of trastuzumab in plasma to trastuzumab in cerebrospinal fluid is > 300:1 [47]. Other several studies highlighted the ability of mABs to reach brain metastases in numerous animal models such as blood-borne tumor from outside the brain, and dormant tumor that grows enough to rupture the BBB, and thus allow mABs to infiltrate [48]. In addition to physical barriers, several functional barriers contribute to the restrictive nature of BBB, which represents a major obstacle to effective drug delivery into the CNS [49]. Future studies should address how these antibodies and drug conjugates display such efficacy in control of CNS tumor burden while movement of antibodies across the BBB is still restricted. Another method for improving treatment, which is currently under investigation, is selectively altering BTB permeability to increase antibody delivery to CNS lesions.

## MATERIALS AND METHODS

### *In-vitro* studies

#### Chemicals and reagents

Texas Red 70,000 MW Dextran (TRD 70 kDa) was purchased from Molecular Probes (Invitrogen, Carlsbad, CA). Trastuzumab (Herceptin^®^, Genentech/ Roche) was buffer-exchanged into 50 mM potassium phosphate buffer and 150 mM sodium chloride adjusted to pH of 6.7. Trastuzumab was fluorescently linked to Rhodamine-123 (Innova Biosciences, Babraham, England). All other chemicals were of analytical grade and purchased from Sigma-Aldrich (St. Louis, MO).

#### Cell culture for *in-vitro* studies

Human Umbilical Vein Endothelial Cells (HUVECs) were purchased from Lonza (Allendale, NJ). CTX-TNA2 rat brain astrocyte cell line was generously provided by the laboratory of Dr. Jim Simpkins (West Virginia University, Morgantown, WV). Both HUVEC and astrocyte line were cultured and maintained in Endothelial Basal Medium – 2 (EBM-2) with the supplementation of EGM-2 Bullet Kits from Lonza (Allendale, NJ). The laboratory of Dr. Patricia Steeg, of the National Cancer Institute, generously provided a JIMT-1 brain metastases of breast cancer cell line, a line which naturally overexpresses HER2. These cells were cultured and maintained in DMEM supplemented with 10% Fetal bovine serum and 1% penstrep. All cell lines for *in-vitro* studies were grown within a 37°C humidified incubator with 5% CO_2_ until ~85-90% confluent.

#### Cell culture in microfluidic chip

The co-culture idealized microvascular microfluidic chips used in this study were obtained from SynVivo Inc (Huntsville, AL). These microfluidic chips were prepared, then cultured with cells and maintained as previously described [37, 38].

#### Transport studies and quantification using fluorescent microscopy

For each device, a BD Leur-lok syringe connected to Tygon tubing was filled with EBM-2 media containing fluorescent tastuzumab. This syringe was then mounted on a programmable Harvard PHD 2000 syringe pump (Harvard Apparatus, Holliston, MA) and tubing was inserted into the device. Chips were maintained at 37°C with 5% CO_2_ and mounted in an automated stage enclosure on a Nikon Eclipse TE2000-E Live Cell Sweptfield Confocal microscope (Melville, NY). Permeability was measured through the perfusion of fluorescently labeled trastuzumab through the outer chamber at 0.1μL/min. Brightfield (25 ms exposure) and TRITC (200 ms exposure) images were acquired every two minutes for 90 minutes with a Photometrics CoolSnap HQ2 Monochrome CCD Camera (Tucson, AZ) with a 20x/0.75 Plan Fluor Phase Contrast objective, having a total field of 6×8 and stitching those images using brightfield with a 10% overlay. Following acquisition, NIS Elements Imaging Software was used to determine Regions of Interest (ROI) and data exported to Prism 6.0. A line of best fit was determined using linear regression (Prism 6.0), and the slope represents the relative rate of accumulation of fluorescence (k_in_) in the central chamber (representing drug concentration found in normal brain) divided by the amount of fluorescence in the outer chamber (representing drug concentration found in the BBB/BTB vasculature). Unless otherwise noted, data are presented as mean ± S.E.M.

#### Kinetic analysis

Unidirectional uptake transfer constants (k_in_) were calculated using the following equation:

$$\left(C_{CC}\;+\;C_{PF}\right)/C_{PF}\;=\;k_{in}\;\left(t\right)\;+\;O_{C}$$ (Equation 1)

Where C_CC_ is the sum intensity of fluorophore in the region of interest in the central compartment (au) at the end of perfusion, C_PF_ is the sum intensity of fluorophore (au) in the region of interest within the outer compartment, t is the perfusion time in minutes from the time the device reached steady state, and O_C_ is the calculated intercept (T = 0 min; “outer compartment volume” (au)). Since the device took 22 minutes to reach steady state, t=0 minutes is 22 minutes after start of the experiment and 0 minutes from the start of steady state. After the determination of a perfusion time where an adequate amount of fluorescent marker was allowed to pass into brain while still remaining in the linear uptake zone, k_in_ was determined [50, 51].

#### Statistical analysis

Using linear regression with best-fit values, the slope of the line (k_in_) was determined. One-way ANOVA analysis, unpaired student t test's with Welch's correction, and an F test to compare variances were used for the comparison of k_in_ values between the unrestricted diffusions, BBB, and BTB models. For all data, errors are reported as standard error of the mean unless otherwise indicated. Differences were considered statistically significant at p < 0.05. (GraphPad Prism version 6.00 for Mac, GraphPad Software, San Diego, CA, USA).

### *In-vivo* studies

#### Chemicals and reagents

Texas red conjugated 625 MW dextran (TRD 625 Da) was purchased from Molecular Probes (Invitrogen, Carlsbad, CA). Trastuzumab (Roche) was buffer-exchanged into 50 mM potassium phosphate buffer and 150 mM sodium chloride adjusted to pH of 6.7. Trastuzumab was radiolabeled with ^125^I. All other chemicals were of analytical grade and purchased from Sigma-Aldrich (St. Louis, MO).

#### Cell culture

Human MDA-MB-231-HER2+ metastatic breast cancer cells expressing enhanced green fluorescent protein (eGFP) and the luciferase construct were cultured in DMEM supplemented with 10% fetal bovine serum and zeocin (300 μg/ml). Cells were harvested at 80% confluency for intracardiac injection. All cell lines were generously provided by the laboratory of Dr. Patricia Steeg at the National Cancer Institute.

#### Experimental brain metastases model

Homozygous Female Nu/Nu (n=20) mice were obtained from Charles River Laboratories (Kingston, NY) and used for all experiments in this study. All animals were 6–8 weeks of age at the initiation of the metastases models and were housed in a barrier facility. All studies were approved by the Animal Care and Use Committee at Texas Tech University Health Sciences Center and conducted in accordance with the 1996 NIH Guide for the Care and Use of Laboratory Animals. Mice were anesthetized with 2% isoflurane and inoculated with 175,000 breast cancer cells in the left cardiac ventricle with the aid of a stereotaxic device (Stoelting, Wood Dale, IL). In this model, previously validated, the inoculum circulates in the peripheral vasculature, arrests in brain capillaries, extravasates across the blood-brain barrier (BBB), and mice develop metastatic lesions predominantly in the brain [29]. After intracardiac injection, mice were placed in a warmed (37 °C) sterile cage and their vitals monitored until fully recovered. Metastases were allowed to develop and visualized with bioluminescent imaging, until neurologic symptoms appeared (~32 days). Animals were then anesthetized with ketamine/xylazine (100 and 8 mg/kg respectively) prior to injection with ^125^I-trastuzumab via IV bolus dose (femoral vein). ^125^I-trastuzumab was allowed to circulate for 24h. TRD 625 Da was injected intravenously (femoral vein), and 10 minutes post-injection, blood samples were obtained. Mice were euthanized via decapitatation.

#### Harvesting of the brain and other tissues and organs

Animals were euthanized, and brain tissue was rapidly removed (less than 60 seconds) and placed in isopentane (-65°C). Brains were sliced (20 μm) using a cryostat (Leica Microsystems, Wetzler, Germany), and sections were mounted on charged gold plated glass slides, air dried, and stored at -80 °C. In addition to the brain, blood and samples from other organs (heart, lungs, liver, spleen, kidney) were collected, washed, and weighed for comparative analysis. Radioactivity was measured immediately following collection (Tri-CARB 2900TR, Perkin Elmer) and expressed as cpm/mg then converted to nCi/g. Distribution ratios are expressed as the amount of radioactivity in the tissue/blood normalized by weight.

#### Quantitative autoradiography (QAR)

Slides were placed in QAR cassettes (FujiFilm Life Sciences, Stamford, CT) along with^125^I autoradiographic standards (Amersham Biosciences). A phosphor screen (FujiFilm Life Sciences, 20×40 super-resolution) was placed on the slides and standards and allowed to develop for up to 14days. QAR phosphor screens were developed in a high-resolution phosphor-imager (FUJI FLA-7000, FujiFilm Life Sciences) and converted to digital images. Digital QAR images were calibrated to^125^I standards and analyzed using MCID Analysis software (InterFocus Imaging LTD, Linton, Cambridge, England). Metastases permeability fold-changes were calculated based on^125^I signal intensity within confirmed metastases locations (determined by eGFP fluorescence image overlays) relative to^125^I signal intensity in normal brain.

#### Fluorescence measurement

Texas red fluorescence was imaged using a DsRed sputter filter (excitation/band λ 545/25 nm, emission/band λ 605/70 nm and dichromatic mirror at λ 565 nm) (Chroma Technologies, Bellows Falls, VT) and eGFP (expressed in MDA-MB-231BR-HER2+) using an ET-GFP sputter filter (excitation/band λ 470/40 nm, emission/band λ 525/50 nm and dichromatic mirror at λ 495 nm) (Chroma Technologies, Bellows Falls, VT). Fluorescence image capture and analysis software (SlideBook 5.0; Intelligent Imaging Innovations Inc., Denver, CO) was used to obtain and quantify fluorescence images. Texas red permeability fold-changes were determined by Texas Red Sum intensity (SI) per unit area of metastases relative to the SI per area of contralateral normal brain regions. If metastases occurred in contralateral regions, adjacent slices containing unaffected tissues of the same brain structure were used as comparative normal brain regions. Tumor area was calculated from regions of interest drawn around each lesion and is reported in mm^2^.

#### Unidirectional uptake transfer constants (k_in_)

All k_in_ values were then calculated from brain distribution volume versus time as previously described [29].

#### Bioluminescent imaging

Mice were injected with D-luciferin potassium salt (150mg/kg; PerkinElmer, Waltham, MA) dissolved in sterile 1X PBS via intraperitoneal (IP) injection and then anesthetized with 2% isoflurane. Fifteen minutes after IP injection of D-luciferin, darkfield images of mice were acquired with an IVIS Lumineer XV (PerkinElmer) to detect bioluminescence. Animals were imaged 1, 3, 6, 9, 12, 24, 48, 72, 96, 120, 144, and 168 hours post intracardiac injection to ensure successful tumor injection and growth.

#### Data analysis

Statistical significance was determined by Student's t-test and one-way ANOVA followed by Bonferroni's multiple comparison's tests. All differences were considered statistically significant at p< 0.05. *In-vivo* data is reported as Mean ± Standard Deviation (SD) unless otherwise noted (GraphPad Prism 7.0, San Diego, CA). Results associated with drug concentration in tumor and brain distant to tumor (BDT) are Mean values of combined readings from all tumor and BDT areas in the study group, without separation by individual animal data. In the case of k_in_ analysis [29], values obtained at individual time points were also pooled together.

## CONCLUSIONS

This model has been previously used to observe small molecule movement and P-glycoprotein efflux [38]. We observed a relatively similar fold increase of trastuzumab *in-vivo* as compared to the *in-vitro* observation in the microfluidic device when comparing fold increases from the BBB to the (BTB). The prediction and evaluation of the ability of various therapeutic and diagnostic moieties to cross the BBB and BTB as well as their brain uptake kinetics are critical to progress efficient brain metastases therapy and diagnosis from basic to translational research. Such knowledge is needed for the early detection and management of high-risk brain metastases in patients. This study demonstrates that, trastuzumab does cross the blood-brain and blood-tumor barriers though probably below efficacious concentrations.

## Abbreviations

BBB: blood-brain barrier
BTB: blood-tumor barrier
CNS: central nervous system
HER2: human epidermal growth factor receptor-2
mAB: monocolonal antibody
QAR: quantitative autoradiography
SI: sum intensity
t-Rho123: trastuzumab-Rhodamine123
